# A randomized controlled trial on the effect of parenting intervention through “Care for Child Development guideline” on early child development and behaviors

**DOI:** 10.1192/j.eurpsy.2023.1025

**Published:** 2023-07-19

**Authors:** R. Kelishadi, O. Yaghini

**Affiliations:** Isfahan University of Medical Sciences, Isfahan, Iran, Islamic Republic Of

## Abstract

**Introduction:**

It is suggested that parenting intervention programs can play a core component in early child development. Given the limited healthcare resources in developing countries, a group design intervention might be cost-effective.

**Objectives:**

Our objective was to assess the effect of parenting interventions in a cost-effective setting through group sessions CCD interventions on children’s development and behaviors in a non-Western, low/middle income country.

**Methods:**

This randomized controlled trial was conducted in a public Pediatrics clinic in Isfahan, Iran. We included 210 pregnant women in their third trimester, and then followed their children for 18 months. The intervention group underwent 5 educational group sessions. The main outcomes were the children’s development and behaviors based on Bayley Scales of Infant and Toddler Development-III (BSCID-III) at 12 months and Children Behavior Checklist (CBCL) at 18-month of age.

**Results:**

Data of 181 children were analyzed (80 in the intervention group and 101 controls. The adjusted median differences were significantly lower in the intervention group than in controls for attention problems (-3.38; SE=1.59; P=0.035), anxiety problems (-2.28; SE=1.03; P=0.007) and pervasive developmental problems (-5; SE= 1.16; P<0.001) based on CBCL results. However, the difference of proportions was not significant in none of the BSCID-III domains in the intervention and control groups.

**Table 1-:** Results of BSID-III in intervention and control groups at 12 months of age ^a^

	**Intervention** **(n=80)** **Median (IQR)**	**Control** **(n=101)** **Median (IQR)**	**P**	**Adjusted median differences** ^**b**^			
				**Coefficient**			**SE**
**Cognitive score**	105 (100-115)	110 (100-120)	0.099	-4.22	2.51	0.095	
**Language score**	112(103-118)	112(103-118)	0.444	-1.92	2.52	0.446	
**Motor score**	103(97-110)	107(91-112)	0.957	-1.09	2.41	0.652

**Conclusions:**

We found that parenting interventions through CCD group sessions were significantly effective on several child’s behavior domains, but not on children’s development. Future longitudinal studies are necessary in this field.

**Disclosure of Interest:**

None Declared

